# Surface plasmon resonance extension through two-block metal-conducting polymer nanorods

**DOI:** 10.1038/s41467-018-03453-z

**Published:** 2018-03-08

**Authors:** Insub Jung, Minkyung Kim, Min Kwak, Geonwoo Kim, Minsun Jang, Sang Min Kim, Doo Jae Park, Sungho Park

**Affiliations:** 1 Department of Energy Science, Sungkyunkwan University, Suwon, 16419 South Korea; 2 Department of Chemistry, Sungkyunkwan University, Suwon, 16419 South Korea; 3 Department of Physics, Hallym University, Chuncheon, 24252 South Korea

## Abstract

Research on surface plasmon resonance coupling of metallic nanostructures is an important area in the field of plasmonics because distinctive collective optical properties can be realized that are different from the individual constituents. Here we report the localized surface plasmon resonance of hybrid metal-organic nanorods. Colloidal-dispersed Au-PPy nanorods were synthesized as a representative material using a modified electrochemical method, and the collective oscillation properties were systematically investigated by comparing these materials with pure Au nanorods. We observed the extended surface plasmon resonance of a hybrid system. The presence of doped-PPy segments on Au segments induced an enhanced coherent electric field due to the partial contribution of π-electrons on the PPy segment, which led to a red-shifted plasmon feature. Additionally, we demonstrated that surface plasmon resonance extension can be tuned by dopant anions, which demonstrates a way of tuning a dopant-induced plasmonic system.

## Abstract (web summary)

Localized surface plasmon resonance can be exploited for a range of applications, but remains difficult to tailor in metal-organic nanostructures. Here the authors synthesize gold-polypyrrole nanorods and observe a unique extended surface plasmon resonance, which they find to be tunable through doping.

## Introduction

Localized surface plasmon resonance (LSPR) is a collective oscillation of conduction band free electrons in metallic nanostructures due to interactions with light. LSPR has been extensively studied in the fields of optical nanoantennas, drug delivery, and biosensing^1,2^. Since LSPR is very sensitive to the shape, size, and surrounding environment (such as dielectric constant), much effort has been devoted to designing a variety of metallic nanostructures (e.g., spheres, rods, tubes, rings) in order to maximize the utilization of LSPR for specific purposes^3−6^. Among these, a one-dimensional nanorod (NR) structure is regarded as an advanced system that allows for interpretation of the spectral and spatial profiles of the SPR due to its unique optical properties. Of primary interest is how SPR behaves in more complex systems (e.g., with a heterogeneous surface morphology or with heterogeneous components)^7–9^ that are different from their single-component counterparts. When two or more nanostructures are separated by a nanoscale gap or connected via a length-limited spacer, the resulting LSPR properties are distinct from those individual nanostructures. This phenomenon has been described as SPR coupling. Research on SPR coupling of metallic nanostructures is an important area in the field of plasmonics because distinctive collective optical properties can be realized that are different from the individual constituents. In this regard, it is of critical importance to investigate SPR coupling to both obtain a fundamental understanding of the material and to allow for the design of potential plasmonic-related devices^10,11^.

Plasmonic coupling of metal–metal nanostructure systems has been explored for a long time, yet tailoring the plasmonic characteristics of metal–organic hybrid nanostructures is still challenging. Especially, metal–organic hybrid systems have long been exploited in diverse fields, including gas sensing, energy storage, and optoelectronics, due to their unique physical and chemical properties^12^. However, there are few papers related to the optical properties of metal–organic NR systems. Many studies have focused on either conjugate polymers as matrices for assemblies with metallic nanoparticles or electronic transport in the form of nanowires^13–15^. Although metal–organic core–shell structures for plasmonic features have been reported by several groups^16,17^, metal core parts are influenced by the shell, which can block some of the incoming light. This makes it difficult to understand the coupling phenomena along the interface between metal and organic components.

Herein we report an experimental investigation of the unique optical properties of metal–organic hybrid NR systems. Our strategy was to generate gold-polypyrrole (Au-PPy) two-segment NRs via modified electrochemical methods. After synthesizing these materials, the optical properties of colloidal-dispersed Au-PPy NRs were systematically investigated by ultraviolet–visible–near infrared (UV-vis-NIR) spectroscopy. We determined the influence of the PPy segment by carefully comparing these Au-PPy NRs with the optical features of single-component Au NRs. We observed an extended SPR, which differed from surface plasmon coupling that originated from the enhanced electric field generated by the metallic gaps. Additionally, we showed that this SPR extension can be tuned by controlling the doping level achieved by dopant anions.

## Results

### Modified electrodeposition method

**Fig. 1:**
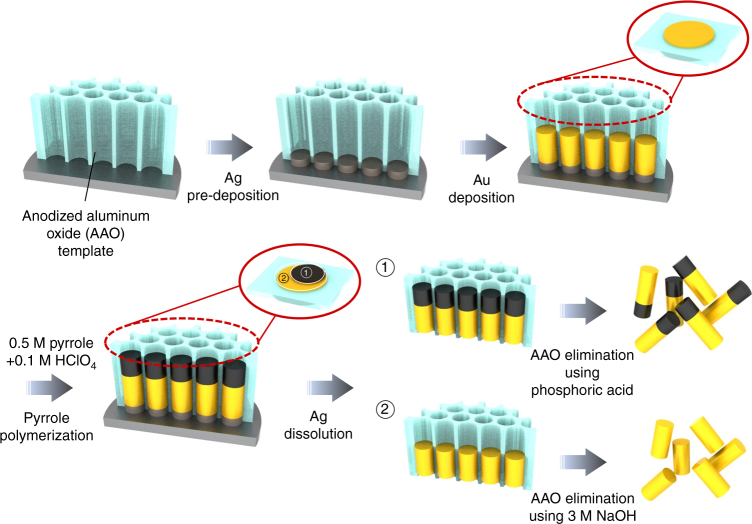
Schematic illustration of the synthesis of Au-PPy NRs O-rings of different sizes were used to separate the Au NR part and the Au-PPy NR part. AAO templates of the Au NR part were dissolved with 3 M NaOH, and AAO templates of the Au-PPy NR part were dissolved with phosphoric acid to prevent de-doping of the PPy segments

**Fig. 2:**
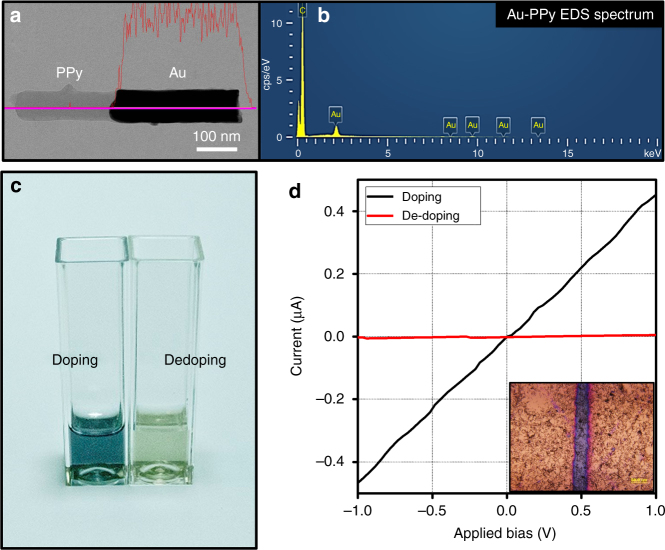
Characterization of as-synthesized Au-PPy NRs. **a** HR-TEM image and EDS line mapping of Au-PPy NRs in which the red line represents the amount of Au atoms. **b** EDS spectrum of Au-PPy NRs that shows the component of Au and C elements. **c** Optical image of PPy NRs under different doping states. When PPy is electro-polymerized, the solution turns into black color. **d** *I*–*V* curve of single-component PPy NRs for doping and de-doping. The inset image shows the PPy NRs (length ~5 µm) randomly dispersed on the Au electrodes

Figure 1 shows a schematic illustration of the synthesis of Au-PPy NRs via an electrochemical deposition method with an anodic aluminum oxide (AAO) template in a three-electrode system. (see Methods section for detailed synthesis). We adopted PPy as a conducting polymer. PPy is conjugated by π-electrons and has been extensively used for energy storage, gas sensing, and electronic devices due to its stability and high conductivity in an oxidative state^13,18,19^. Controlling the length is critical for spectral profile comparisons of Au-PPy NRs and pure Au NRs because even small variations in length can induce a change in the plasmon band (see Supplementary Fig. 1). This is due to the fact that the plasmon features of NRs are normally governed by aspect ratio (the ratio of the diameter to the length of the NR). Thus the Au segment was deposited first, and PPy segments were grown separately so that the effect of the PPy segment could be isolated. AAO templates containing only Au segments were dissolved in 3 M NaOH. AAO templates with Au-PPy NRs were treated with H_3_PO_4_, which leads to simultaneous release of the NRs and protonation of the PPy segments. It should be noted that a natural sink process is needed during each centrifuge washing steps in order to reliably measure optical extinction of colloidal-disposed NRs synthesized by electrochemical method with AAO templates. Without this process, due to a loss of analytes, it is hard to measure optical properties of NRs using UV-vis-NIR spectroscopy. Using this modified electrodeposition method, we were able to systematically investigate the optical properties of Au-PPy NRs dispersed in D_2_O, all of which could be monitored by UV-vis-NIR spectroscopy. A high-resolution transmission electron microscopic image of representative Au-PPy NRs (Fig. 2a) displays well-defined junction between Au and PPy segment without breakage. Each component (Au and C elements) was confirmed by an energy-dispersive X-ray spectroscopic spectrum (Fig. 2b). The doping and de-doping processes used for the PPy segment were further confirmed by optical image (Fig. 2c). When pyrrole is polymerized, PPy is black in color and the color disappears after de-doping. In order to make sure doping/de-doping of PPy before the study of optical properties of Au-PPy NRs, we also performed the electrical measurements for PPy NRs before/after de-doping by immersing those in 3 M NaOH for 7 h. A SiO_2_ wafer was used as a back gate and Au electrodes were deposited (channel length ~40 µm). PPy NRs before/after de-doping were randomly dispersed on the Au electrodes and the *I*–*V* curve was plotted in Fig. 2d as indicated by the slope and the amount of current, de-doped PPy samples did not show the conductivity as indicated by the slope as well as the amount of current (red curve) due to the attenuated electron pathway after de-doping, which indicates the successful doping/de-doping process.

### SPR extension

**Fig. 3:**
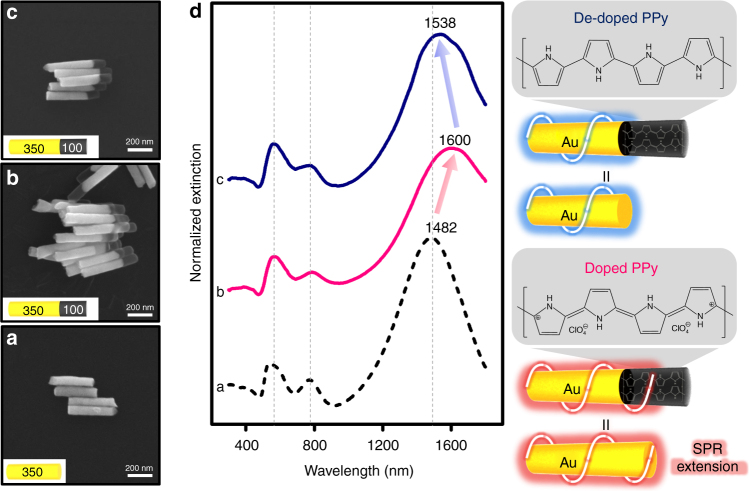
Surface plasmon resonance extension FE-SEM images of **a** single-component Au NRs, **b** doped Au-PPy NRs, and **c** de-doped Au-PPy NRs. The insets show the dimensions of each block, with a bright block for Au and a dark block for PPy. The numbers represent the length in nanometers. **d** Corresponding UV-vis-NIR extinction spectra. The illustrations in the right panel describe the surface plasmon resonance extension under doping/de-doping of the PPy segment

The length of the Au segment in Au-PPy NRs was fixed to 350 ± 15 nm and the diameter is 80 ± 5 nm that is solely dependent upon the pore size of AAO templates (Fig. 3a) and its corresponding UV-vis-NIR spectrum is shown as spectrum a in Fig. 3d. Au NRs in this size regime show three plasmon peaks^20,21^. The peak at 543 nm can be assigned to the dipole transverse mode (electrons oscillating along the short axis of NRs), and the peak at 1482 nm indicates the dipole longitudinal plasmon band (electrons oscillating along the long axis of NRs). The peak at 774 nm is a quadrupole plasmon mode that occurs due to the phase retardation when the length of the Au NRs is over a certain limit (~250 nm with 80 nm in diameter), which is a quality factor for ensuring the homogeneity of the synthesized Au NRs. Based on this, we fabricated doped Au-PPy NRs with a 100 ± 5 nm PPy segment, as shown in Fig. 3b, and its corresponding spectrum is shown as spectrum b in Fig. 3d. Compared to pure Au NRs, the transverse plasmon mode of the Au-PPy NRs exhibited a negligible plasmon shift (Δ*λ*). Interestingly, the dipole longitudinal plasmon band was substantially red-shifted from 1482 nm to 1600 nm, which is an optical feature that is similar to the pure Au NRs with comparable dimensions (~450 nm). This is contrary to what we expected of the Au-PPy NRs. Specifically, we expected that both plasmon bands would be blue-shifted or that we would observe peak broadening in the transverse plasmon band since PPy generally absorbs strongly in the UV-vis region (around 460 nm). In our previous works, optically less active Ni metal segments served as a bridge to effectively couple the SPR between Au segments at the end by providing free electrons^20^. However, PPy has no surface plasmon and only limited movement of π-electrons in the polymeric chains (see Supplementary Fig. 2). Under a doping state (p-doping in the case of PPy), the formation of spin-less bipolaron bands generates mid-gap states (this occurs under a high doping level as in our case, i.e., over 25 mol%) between the electronic band structures in PPy^22,23^. When an interface between the Au and the PPy segment is formed, the Fermi level drops. Localized mobile bipolarons (charge carriers) of PPy are strongly influenced by the free electrons of Au blocks, and they hop into the conduction band of Au. In other words, when free electrons on the Au NRs absorb energy, they strongly oscillate on the Au NR surface. Then the movement of π-electrons on the PPy blocks is mediated by the coherent oscillation from the conduction-free electrons on the Au surface, which leads to a surface plasmon coupling effect. Thus the oscillation of Au free electrons facilitates charge transfer, and the electromagnetic wave propagates over the Au-PPy junction, which results in an extended SPR.

To verify our hypothesis, we synthesized de-doped Au-PPy NRs by immersion in a 3 M NaOH solution for 7 h (Fig. 3c). As shown in the corresponding spectrum (spectrum c, blue trace, Fig. 3d), quadrupole and diploe longitudinal plasmon bands exhibited blue-shifting of 13 nm and 63 nm, respectively, compared to those of doped-state NRs, which were quite similar to those in the spectrum of the original Au NRs. During the de-doping process, π-electrons in PPy segments were interrupted by hydroxyl groups, which hindered the coherent oscillation of free electrons on the Au NR surfaces. In other words, a lower concentration of perchlorate ions produced two polarons (less favorable than bipolarons) that recombined. This recombination prevented an optical transition and therefore displayed similar optical behavior to that of the original Au NRs. The slight red-shift (1538 nm) in the de-doped Au-PPy NRs compared to the original pure Au NRs (1482 nm) might be attributed to the change in the ionic strength of the solution during subsequent washing with deionzied (DI) water^24,25^. If there were aggregation of NRs, transverse plasmon band would be shifted with much broader band widths since the aspect ratio of NRs would be totally changed. However, considering well-defined transverse plasmon band and stability over 2 h (Supplementary Fig. 3), this indicates that the aggregation is not the major factor that generate the longitudinal plasmonic shifts.

**Fig. 4:**
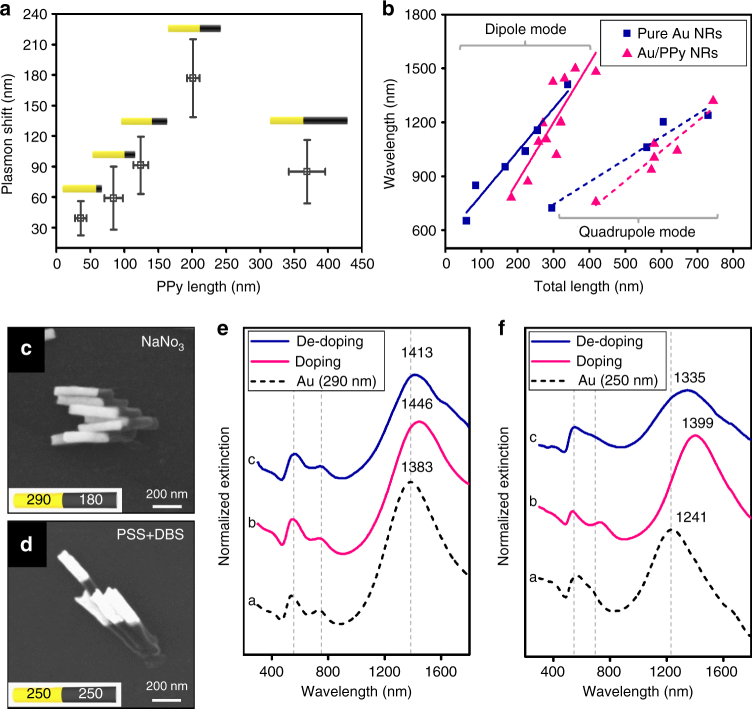
In-depth study of surface plasmon resonance extension. **a** Plasmon shift of Au-PPy NRs with respect to pure Au NRs as a function of lengths of PPy segments. All plasmon shifts was measured at doped state. The error bar represents a standard deviation from three measurements from different nanorods batches. **b** Extension depths measurement of pure Au NRs and Au-PPy NRs for dipole and quadrupole plasmon modes. FE-SEM images of Au-PPy NRs with dopants: **c** NaNO_3_, and **d** poly(sodium 4-styrenesulfonate) with sodium dodecylbenzenesulfonate. **e**, **f** Corresponding UV-vis-NIR extinction spectra of Au-PPy nanorods with different dopant anions. UV-vis-NIR spectra (**e**, **f**) were corresponding to Au-PPy nanorods shown in **c**, **d**. Each dashed trace represents the extinction spectrum of pure Au NRs with the given lengths

From the above analysis, the extension is generated mainly across the interface between the metal and polymer blocks upto a certain length in PPy segments. Since the population density of the charge carrier (in organic conjugates) is much lower than that of free electrons in metallic structures, only a partial contribution occurs, giving rise to extended SPR instead of intra-particle SPR coupling. To correlate the SPR extension with the length of the PPy segments, we measured plasmon shift as a function of PPy lengths with Au NRs (275 ± 25 nm) (Fig. 4a). The linear relationship between the plasmon shift and PPy segments can be confirmed within the range up to 200 nm of PPy segments, which indicates that SPR extension can be effectively operative up to 200 nm. Beyond this range (ca. *L* > 200 nm), however, the PPy length does not significantly affect plasmonic shift, rather showing inconsistent plasmonic shift attributed to the partial agglomeration of NRs due to the strong van der Waals interactions among PPy segments. We also measured the extension depths of pure Au NRs and Au-PPy NRs (by fixing the length of PPy segments ~80 ± 5 nm) for dipole and quadrupole plasmon modes (Fig. 4b) as a function of total length (i.e., by only increasing Au block lengths). The slopes (Δ*λ*/*L*) of Au-PPy NRs for both of dipole and quadrupole modes exhibited larger slopes than the case with pure Au NRs (dipole mode: 3.3 vs. 2.4, quadrupole mode: 1.7 vs. 1.3, respectively). It indicates that the presence of PPy segment induces the perturbation into the system, endowing the NRs with much higher sensitivity toward the change of environment, as compared to the case of pure Au NRs. This phenomenon implies that a small portion of conducting polymer block can significantly modulate the collective optical features and provide additional options for controlling the plasmonic characteristics (e.g., immunoassay using plasmonic NRs where sensitivity of nanoprobes is the key factor, which are underway).

### SPR extension controlled by dopant anions

It is well known that the physical properties of conducting polymers, including PPy, are strongly modified by the dopant anion used during electrochemical polymerization. Along these lines, the electrical performance of PPy films with a variety of dopants has been described in terms of the conductivity of anions^26^. Not only the proportion and length of the PPy segments but also an alternative strategy for tailoring the plasmon extension is highly desirable for potential applications. For that reason, we synthesized Au-PPy NRs with different dopant anions in order to correlate the plasmon resonance extension with the conductivity of the dopants. As a proof-of-concept, we employed NaNO_3_ and poly (sodium _4_-styrenesulfonate) with sodium dodecylbenzenesulfonate (PSS/DBS) as dopants. The corresponding FE-SEM images of Au-PPy NRs are shown in Fig. 4c, d (Au(290 ± 25 nm)–PPy(180 ± 10 nm) in Fig. 4c, Au(250 ± 12 nm)–PPy(250 ± 15 nm) in Fig. 4d). We observed red-shifting of longitudinal modes for all two samples around Δ*λ* = 63 and 158 nm relative to their pure Au components, respectively (Fig. 4e, f). When inorganic NO_3_^−^ anions were used, the plasmon shift was almost half of that observed for ClO_4_^−^ in Fig. 3 (Δ*λ* changed from 118 nm to 63 nm). This can be attributed to the difference in anion conductivity. To support this, we used organic molecules as dopants (PSS/DBS), the conductivity of which is known to be much higher than that of NO_3_^−^ anions^26^. In this case, the plasmon shift increased to 158 nm when PSS/DBS were used as dopants (Fig. 4f). This is due to the π–π interactions between organic anions and aromatic pyrrole rings, which facilitated the enhanced electron oscillation pathways^27^. It is noteworthy that this SPR extension can be tuned by controlling the dopant level, indicating that this system employs an electronic transport mechanism^28^. Since there are a variety of conducting polymers with diverse anions, dopant-induced tuning of plasmonic excitation will become an interesting topic for plasmonic-related applications. To rule out the effect of pH, all analyses were performed with a pH value around 7 (NaNO_3_: 7.74, PSS/DBS: 7.19).

### Refractive index effect on SPR extension

**Fig. 5:**
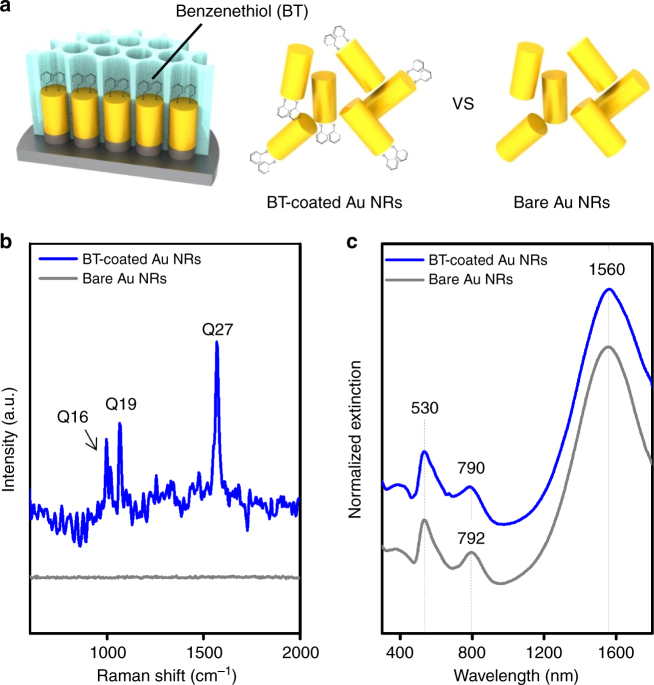
Refractive index effect on surface plasmon resonance extension. **a** Schematics for functionalization of benzenethiol on top of one-side Au NRs. **b** Raman spectra for Au NRs with/without benzenethiol coating. Raman peaks are assigned as mode Q16: S-H bending at 996 cm^−1^, Q19: C-S stretching at 1065 cm^−1^, and Q27: C-C stretching at 1568 cm^−1^. **c** Corresponding UV-vis-NIR extinction spectra

**Fig. 6:**
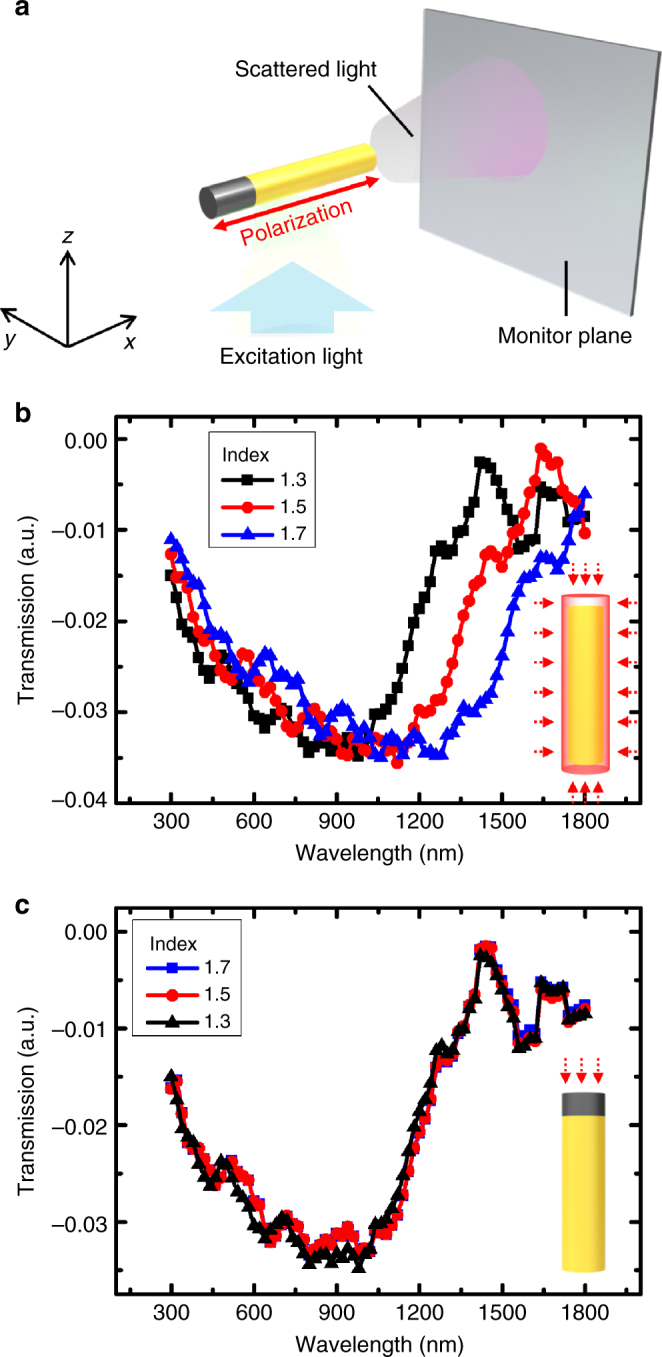
FDTD method for limited alteration of refractive index. **a** Schematic illustration of simulation design. **b** Extinction spectra of Au NRs for different indices of the surrounding media of 1.3, 1.5, and 1.7. **c** Extinction spectra of Au-PPy NRs with regard to the refractive index of the PPy segment while the refractive index of the surrounding was fixed as 1.3

Of critical interest here is whether or not the plasmon extension phenomenon arises from the change in the dielectric constant as the PPy segment is introduced into the system. There are several reports about Au–polymer core–shell nanostructures that demonstrated that the change in optical properties was mainly due to the change in refractive index induced by the polymer shell^29–31^. However, it is questionable whether that reasoning can be applied in our case where the PPy segment enclosed only one side of the Au segment, while the rest of the Au block was directly exposed to the light. In that context, one side of the pure Au NRs (355 ± 20 nm in length) was modified with benzenethiol (BT) to produce a configuration similar to that of Au-PPy NRs (Fig. 5a). We observed three typical Raman peaks of BT (blue line in Fig. 5b), which indicate the successful modification of the top Au surface and the presence of BT. Three Raman peaks are assigned as C-C stretching at 1568 cm^−1^, C-S stretching at 1065 cm^−1^, and S-H bending at 996 cm^−1^ (see ref. ^32^ for further detail of Raman analysis of BT). Notably, the entire plasmon bands of both BT-capped Au NRs and unmodified Au NRs were nearly identical (Fig. 5c), which implies that limited alteration of the dielectric constant does not affect SPR. To further take into account comparable length modifications with PPy, we performed similar experiment with polyethylene glycol (PEG; *M*_w_: 6000, average chain lengths ~60.5 nm) and observed that there were little change of plasmonic change in case of only tip-modified Au NRs, in contrast to the obvious plasmonic change in case of whole surface modification (Supplementary Fig. 4). This was further supported by theoretical simulation by a finite difference time domain (FDTD) method (see Fig. 6a for schematic illustration of modeling and see Methods for further details). Figure 6b depicts the extinction spectra for different indices of the surrounding media of 1.3, 1.5, and 1.7, with polarization parallel to the long axis of NRs (see also Supplementary Fig. 5 for the extinction spectra for different indices of the imaginary part). It is clear that the resonance associated with a localized surface plasmon polarition (LSPP) excitation near 1.4 μm shifts to the longer wavelength as refractive index increases. Such red shift is obviously due to the elongation of the effective length of the NR due to refractive index change of the surrounding. In contrast, when the refractive index of the polymer was attached and changed from 1.5 to 1.7, while fixing the refractive index of the surrounding as 1.3, no significant change in spectra was observed, as depicted in Fig. 6c. Such behavior indicates that the variation of the polymer, having limited volume fraction, hardly affects the resonance condition of the LSPP. Therefore, the change of the dielectric constant caused by the incorporation of the PPy segment onto one side of Au segment is not enough to induce the observed plasmon shift. Conclusively, the spectral profile variation in this work was caused not by a refractive index change but mostly by π-electrons that partly contribute to the collective oscillation of free electrons in the Au segment. As a control, extinction spectra calculated under polarization parallel to the short axis of NRs was calculated, showing no peak changes due to less sensitive nature of transverse plasmon modes (Supplementary Fig. 6).

### pH-dependent optical property of Au-PPy NRs

**Fig. 7:**
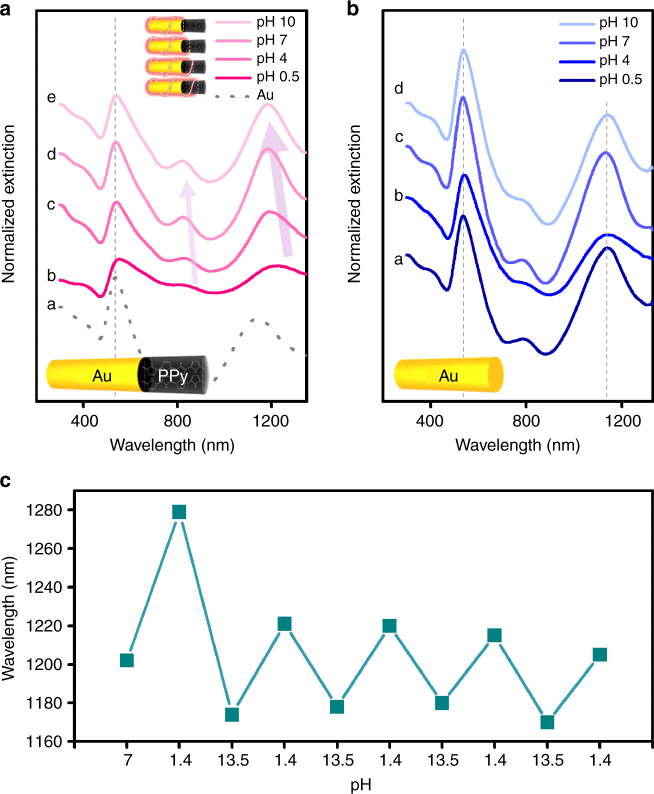
pH-dependent optical property of Au-PPy nanorods UV-vis-NIR spectra of **a** Au-PPy NRs and **b** pure Au NRs as a function of pH-value from 0.5 to 10. **c** Reversibility measurement of Au-PPy NRs as the solution pH was cycled. The plot shows the dipole mode vs pH variations

Finally, the protonation/deprotonation of Au-PPy NRs was measured by real-time monitoring of plasmon band shifting as a function of pH (from 0.5 to 10) (Fig. 7a). The pH-dependent optical measurement was performed with Au-PPy NRs (550 ± 20–100 ± 10 nm) by serially mixing the base solution into the acid solution in one batch while monitoring the system using UV-vis-NIR spectroscopy. As the pH value gradually changed from pH 0.5 to 4, 7, and 10, quadrupole longitudinal plasmon bands were incrementally blue-shifted from *λ* = 1225 to 1190, 1187, and 1183 nm (Δ*λ* = 42 nm), respectively, in accordance with the aforementioned analysis. These results indicate the successful modulation of plasmonic properties of Au-PPy hybrid NR structures. We also observed a systematic change in the octupole higher-order plasmon mode (from *λ* = 836 nm at pH 0.5 to *λ* = 818 nm at pH 10). The modulated higher-order modes included the quadrupole mode, and these results suggest that SPR extension can take place regardless of plasmon mode. As a control group, pure Au NRs were also measured under the same conditions (Fig. 7b). Neither the quadrupole nor the octupole longitudinal modes of pure Au NRs changed with variations in pH. Reversibility measurement was also performed (Fig. 7c) up to five cycles of the solution pH variations (Au (240 ± 12 nm)–PPy (88 ± 10 nm) NRs). As the cycles proceeded, the degree of plasmon shift slightly decreased (which is due to the hysteresis of doping and dedoping processes), finally reaching 1200 nm without agglomeration of Au-PPy NRs, suggesting that this hybrid nanostructures can be utilized in active plasmonic switching devices.

In conclusion, we report the plasmonic properties of hybrid metal–organic (Au-PPy) two-segment NRs via an electrochemical deposition method with the assistance of AAO templates. Using a modified synthetic method, we systematically investigated the optical properties of Au-PPy NRs by UV-vis-NIR spectroscopy. We observed an extended SPR caused by the partial contribution of π-electrons in PPy segments to the coherent oscillation of conduction-free electrons in Au segments. We further confirmed this by controlling the doping level of PPy segments and showed that the plasmon resonance extension can be tuned by using different dopants, which paves the way for plasmonic modulation. We expect that this study will contribute to the fundamental understanding of plasmonic coupling effects and will find applications in optoelectronics and plasmonic nanoantennas.

## Methods

### Synthesis of Au and Au-PPy NRs

Segmented Au-PPy hybrid NR structures were synthesized via a template-directed potentiostatic electrochemical deposition technique. A homemade AAO template was used throughout the experiments. The detailed synthesis of this is described elsewhere^33^. First, a silver layer (up to 400 nm thick) was evaporated on one side of the AAO template, and this served as a working electrode in a three-electrode electrochemical set-up after making physical contact with a glassy carbon electrode in the Teflon cell. A three-electrode configuration was formed with a Ag/AgCl reference electrode and a Pt wire counter electrode. In the pre-deposition step, the gap between the AAO and the conducting layer was filled with Ag (Technic ACR silver RTU solution from Technic Inc.) at −0.95 V. Au was electroplated on the surface using an Orotemp 24 RTU (from Technic Inc.) at −0.95 V. Au block length was controlled using the total charge that passed through the cell. PPy was electrochemically polymerized by sweeping the potential between −1.2 V and 0.7 V at a scan rate of 0.1 V s^−1^ and sweeping the potential between −1.2 V and 0.9 V at a scan rate of 0.1 V s^−1^. The length of PPy was controlled by changing the number of scan cycles. A homemade monomer solution (0.5 M pyrrole from Sigma-Aldrich with 0.1 M HClO_4_) was used for the electro synthesis of PPy. To separate the Au NRs part and the Au-PPy NRs part, the Au block was deposited using an O-ring with a 1 cm diameter, and the PPy block was deposited using an O-ring with a 0.7 cm diameter. The Ag layer was etched out using nitric acid. Au NRs were removed from the AAO using 3 M sodium hydroxide solution. Au-PPy NRs were removed from AAO using phosphoric acid (from Samchun Chemical). The resulting samples were rinsed with distilled water. The sample was dispersed in D_2_O and was then characterized using a UV-vis-NIR spectrophotometer (UV-3600 spectrophotometer, Shimadzu). All the UV spectra are normalized with respect to the transverse surface plasmon mode in order to compare and interpret the dipole/quadrupole plasmon modes. The spectra have also been vertically offset for clarity. Importantly, for each and every washing step, 6 h of waiting period was allowed so that samples could be naturally sunk to the bottom of Eppendorf tubes. In detail, AAO templates with Au NRs or Au-PPy NRs after elimination of Ag pre-deposition were inserted into Eppendorf tube and dispersed in NaOH or phosphoric acid solution. After elimination of AAO templates, NRs were released into the solution. After centrifugation (5000 rpm, 50 min) of the solution, NaOH or phosphoric acid was extracted. Then DI water was poured into the tubes, followed by mild sonication. After several hours, centrifugation (5000 rpm, 60 min) were performed, and again DI water was extracted with new DI water injection. Importantly, without a waiting process before/after centrifugation of NR solution, there is a loss of samples not enough to measure optical properties. This applies to all the optical measurements for samples synthesized by electrochemical deposition.

### Synthesis of de-doped Au-PPy NRs

The PPy block was de-doped using 3 M sodium hydroxide for 7 h. The resulting samples were re-dispersed and rinsed with distilled water, and UV-vis-NIR extinction measurements were performed in D_2_O.

### Synthesis of BT/PEG-modified on top of Au surface

Au NRs deposited within AAO templates were treated with 0.1 M BT for 1 h, and then sequential elimination of Ag pre-deposited layers and AAO templates was performed, which led to Au NRs with BT caps on top of the Au NRs. In case of PEG-modified on top of Au surface, Au NRs deposited within AAO templates were immersed in 1 mM PEG for 2 h, followed by DI washing and the elimination of Ag pre-deposited layers and AAO templates, respectively. These samples were dispersed onto SiO_2_ wafer for Raman measurement (WITEC alpha300, 532 nm excitation, acquisition time: 10 s).

### COMSOL modeling

Simulation extracting extinction spectra of NR was performed by using an FDTD method. As depicted in Fig. 6a, light in the form of plane wave is incident from the side of the NR, with polarization parallel to the long axis. This light source was positioned at 1 μm apart from the NR, with size of 3 μm in *x* direction and 2 μm in *y* direction. The emission direction was along *z* axis with polarization direction along *x* axis. The NR was positioned at the long axis, which is along the *x* axis, having diameter of 80 nm and length of 350 nm. When the polymer with the length of 100 nm was attached, total length of the nanorod+polymer assembly was 450 nm. Scattered light was collected by a monitor in *yz*-plane, positioned 1 μm apart from the nanorod with the size of 1 μm in *y* axis and 2 μm in *z* axis. In case of the polymer attachment, monitor was positioned the opposite side of the polymer, to avoid the effect of multiple reflections and corresponding distortion of the spectra. Grid size was 8 nm near the nanorod and 45 nm in the other side. Simulation volume was 3 μm in *x* axis, 1 μm in *y* axis, and 4 μm in *z* axis and was assumed to be filled with the dielectric media, having refractive index of 1.3, 1.5, and 1.7. The perfectly matched layer was applied at the border.

### Data availability

The authors declare that all data are available from the corresponding authors on reasonable request.

## Electronic supplementary material

Supplementary Information
