# Supplementary material for: Surface plasmon resonance extension through two-block metal-conducting polymer nanorods
Source: Nat Commun. 2018 Mar 8;9:1010. doi: 10.1038/s41467-018-03453-z (PMC5843636; doi:10.1038/s41467-018-03453-z)
Supplement: Supplementary file 1 — Supplementary Information [file 41467_2018_3453_MOESM1_ESM.pdf]

Surface Plasmon Resonance Extension through Two-Block Metal-Conducting Polymer Nanorods

Insub Jung et al.

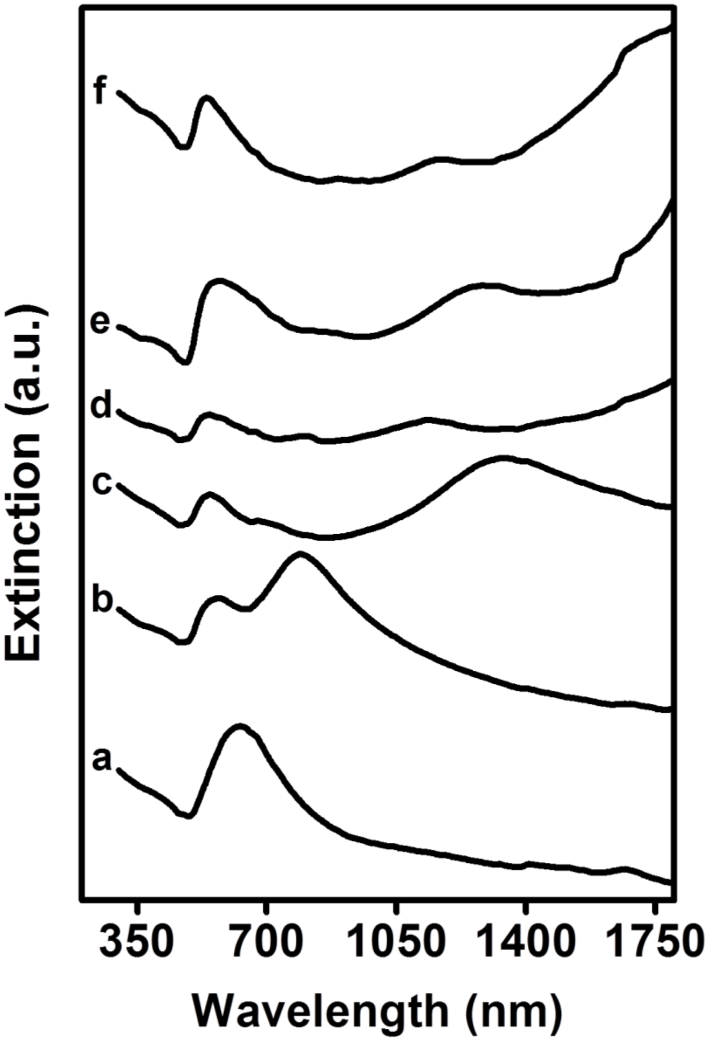

Supplementary Figure 1. UV-vis-NIR spectra of pure Au NRs as a function of lengths. (a) 65±5 nm, **(b)** 122±11 nm, **(c)** 288±20 nm, **(d)** 600±50 nm, **(e)** 720±32 nm, **(f)** 850±55 nm. As the lengths of Au NRs increase, both dipole and quadrupole plasmon bands redshift to longer wavelengths, contrast to negligible shifts of transverse plasmon mode that is insensitive to external environments.

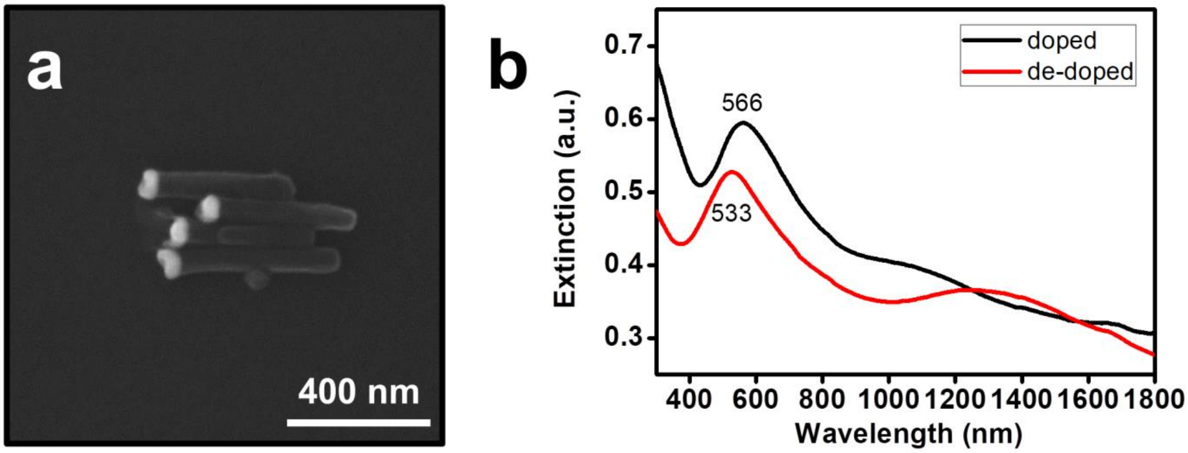

Supplementary Figure 2. (A) FE-SEM image of Au-PPy NRs **(B)** Corresponding UV-vis-NIR spectra for doped and de-doped Au-PPy NRs. Single-component PPy NRs were not stable in its nanorods form thus, we incorporated disk-like thin conductive Au segments in order not only to deposit PPy segment, but also to produce stable colloidal solution for appropriate optical measurements. As expected, both doped and de-doped Au-PPy NRs with this dimension showed no specific absorbance within the range where surface plasmon resonance extension takes place we are interested in, except plasmon peaks from Au disk. It is noteworthy that after de-doping process, plasmon peak was blue-shifted from 566 nm to 533 nm, which indicates that surface plasmon resonance extension occurs only under doped PPy states.

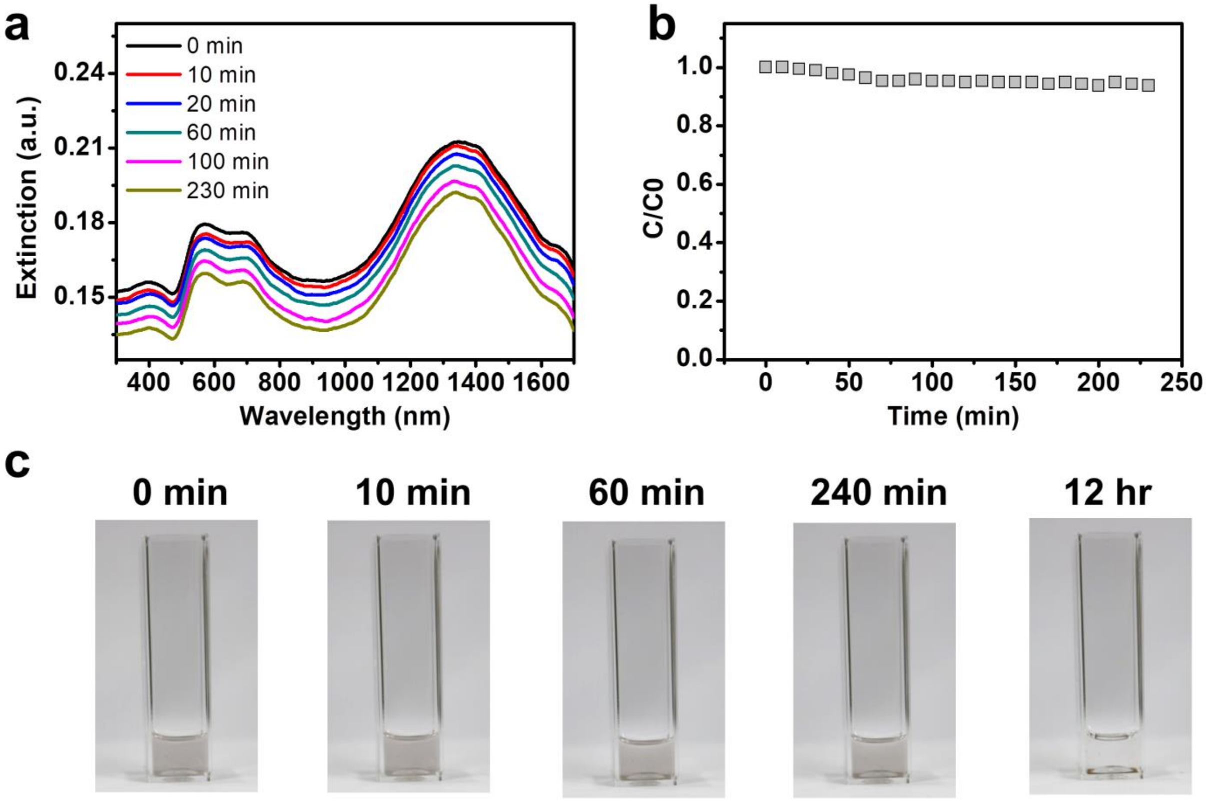

Supplementary Figure 3. (a) Time-dependent monitoring of UV-vis-NIR spectra of Au NRs (260±20 nm). **(b)** Kinetic assay of Au NRs as a function of time measured at 1340 nm. **(c)** Optical photographs of Au NRs solution as a function of time. Only after 12 hr, Au NRs totally sunk down on the bottom of the cuvette.

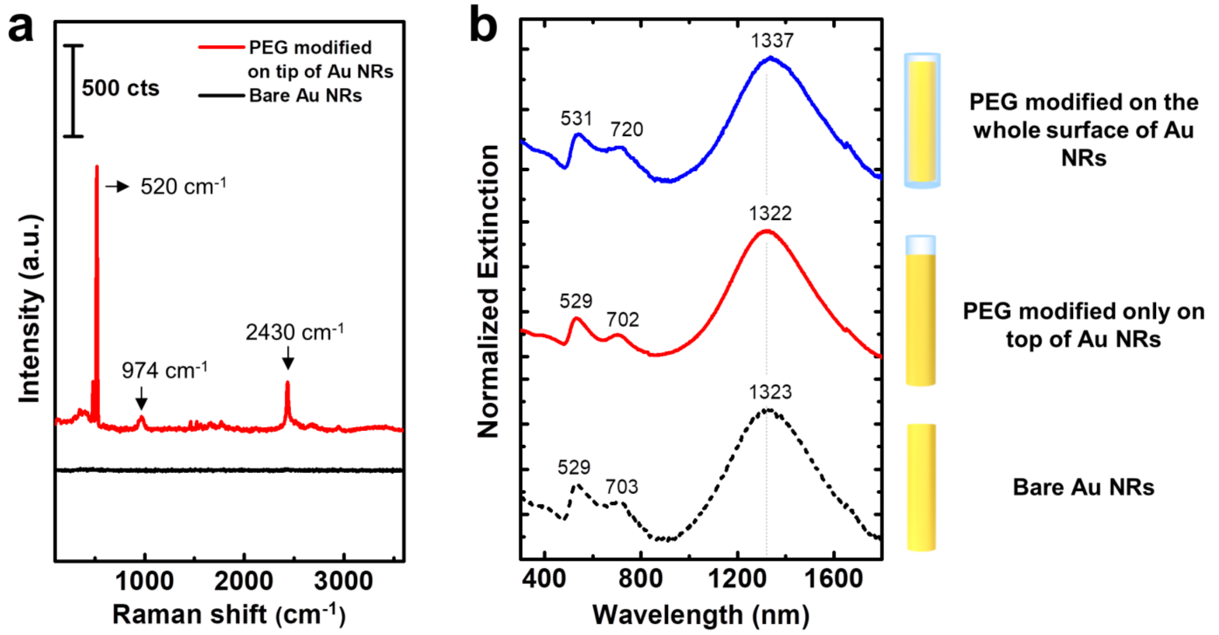

Supplementary Figure 4. Refractive index effect on surface plasmon resonance extension. (a) Raman spectra for Au NRs (265±15 nm) functionalized with PEG on top of one-side Au NRs (red line) and for Au NRs without PEG coating (black line). Raman peaks are assigned as the C-C-O bending vibration at 520 cm^{-1}, the C-O stretching at 974 cm^{-1}, the symmetric stretching vibrations of methylene group of PEG at 2430 cm^{-1}, respectively[1]. **(b)** Corresponding UV-vis-NIR extinction spectra.

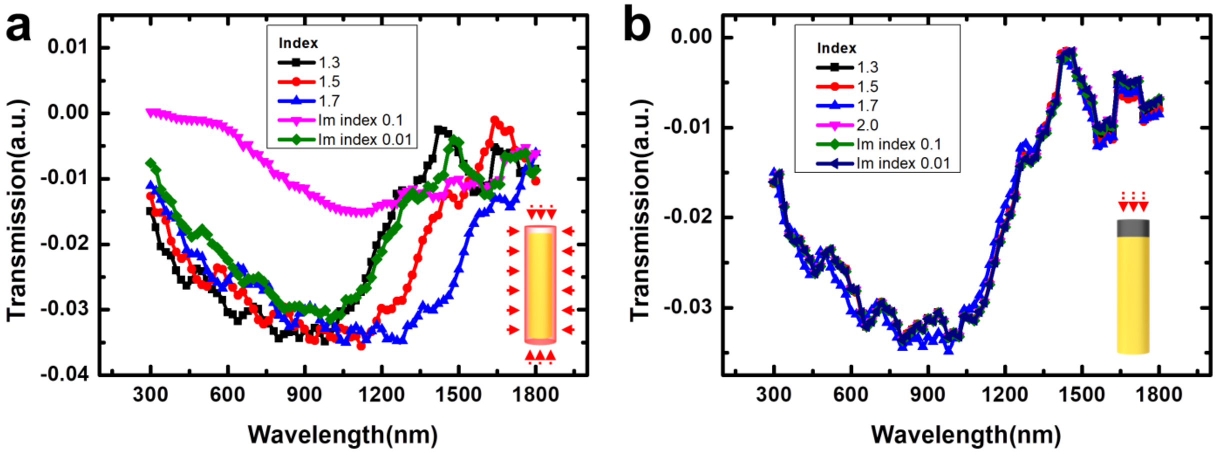

Supplementary Figure 5. FDTD method for limited alteration of refractive index. (a) Extinction spectra of Au NRs for different indices of the surrounding media of 1.3, 1.5, 1.7 and extinction spectra of Au NRs for different imaginary part of refractive index unit (0.1 and 0.01). **(b)** Extinction spectra of Au-PPy NRs with regard to the refractive index of the PPy segment while the refractive index of the surrounding was fixed as 1.3. Extinction spectra of Au-PPy NRs with regard to the refractive index with imaginary part (0.1 and 0.01) were also plotted.

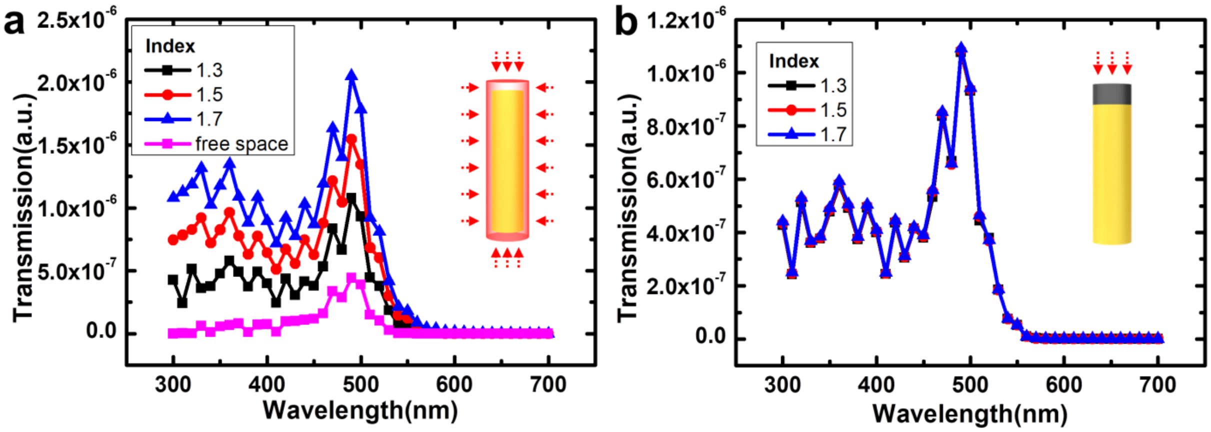

Supplementary Figure 6. FDTD method for limited alteration of refractive index. (a) Extinction spectra of Au NRs for different indices of the surrounding media of 1.3, 1.5, and 1.7 under polarization parallel to the short axis of the NRs. **(c)** Extinction spectra of Au-PPy NRs with regard to the refractive index of the PPy segment while the refractive index of the surrounding was fixed as 1.3 under polarization parallel to the short axis of the NRs.

Supplementary Reference

[1] Yamini, D., Venkatasubbu, G., Kumar, J. & Ramakrishnan, V. Raman scattering studies on PEG functionalized hydroxyapatite nanoparticles. *Spectrochimica Acta Part A: Molecular and Biomolecular Spectroscopy* **117**, 299-303 (2014).
